# Analyzing the emerging patterns of SARS‐CoV‐2 Omicron subvariants for the development of next‐gen vaccine: An observational study

**DOI:** 10.1002/hsr2.1596

**Published:** 2023-10-18

**Authors:** Ranjan K. Mohapatra, Snehasish Mishra, Venkataramana Kandi, Francesco Branda, Azaj Ansari, Ali A. Rabaan, Md. Kudrat‐E‐Zahan

**Affiliations:** ^1^ Department of Chemistry Government College of Engineering Keonjhar Odisha India; ^2^ School of Biotechnology, Campus‐11 KIIT Deemed‐to‐be‐University Bhubaneswar Odisha India; ^3^ Department of Microbiology Prathima Institute of Medical Sciences Karimnagar Telangana India; ^4^ Department of Computer Science, Modeling, Electronics and Systems Engineering (DIMES) University of Calabria Rende Italy; ^5^ Department of Chemistry Central University of Haryana Mahendergarh Haryana India; ^6^ Molecular Diagnostic Laboratory Johns Hopkins Aramco Healthcare Dhahran Saudi Arabia; ^7^ College of Medicine Alfaisal University Riyadh Saudi Arabia; ^8^ Department of Public Health and Nutrition The University of Haripur Haripur Pakistan; ^9^ Department of Chemistry Rajshahi University Rajshahi Bangladesh

**Keywords:** COVID‐19, next‐gen vaccine, Omicron variants, pandemic tracking, SARS‐CoV‐2 variant, vaccine efficacy

## Abstract

**Background and Aim:**

Understanding the prevalence and impact of SARS‐CoV‐2 variants has assumed paramount importance. This study statistically analyzed to effectively track the emergence and spread of the variants and highlights the importance of such investigations in developing potential next‐gen vaccine to combat the continuously emerging Omicron subvariants.

**Methods:**

Transmission fitness advantage and effective reproductive number (*R*_e_) of epidemiologically relevant SARS‐CoV‐2 sublineages through time during the study period based on the GISAID data were estimated.

**Results:**

The analyses covered the period from January to June 2023 around an array of sequenced samples. The dominance of the XBB variant strain, accounting for approximately 57.63% of the cases, was identified during the timeframe. XBB.1.5 exhibited 37.95% prevalence rate from March to June 2023. Multiple variants showed considerable global influence throughout the study, as sporadically documented. Notably, the XBB variant demonstrated an estimated relative 28% weekly growth advantage compared with others. Numerous variants were resistant to the over‐the‐counter vaccines and breakthrough infections were reported. Similarly, the efficacy of mAB‐based therapy appeared limited. However, it's important to underscore the perceived benefits of these preventive and therapeutic measures were restricted to specific variants.

**Conclusion:**

Given the observed trends, a comprehensive next‐gen vaccine coupled with an advanced vaccination strategy could be a potential panacea in the fight against the pandemic. The findings suggest that targeted vaccine development could be an effective strategy to prevent infections. The study also highlights the need of global collaborations to rapidly develop and distribute the vaccines to ensure global human health.

## 1 INTRODUCTION

Within days of its emergence, the World Health Organization (WHO) designated Omicron virus variant as the fifth variant of concern (VOC) after alpha, beta, gamma, and delta. More than 200 lineages/sublineages have emerged since then.^1^, ^2^ As a result of the accumulating mutations in the replicating virus triggered by the host's immune pressure, Omicron VOC demonstrates large diversity. BA.1, BA.2, and BA.3 of these variants were responsible for the fourth wave of the epidemic.^3^ New lineages BA.4 and BA.5 emerged quickly thereafter displacing the earlier ones.^4^ In July 2022 the sublineage BA.2.75, nicknamed as “Centaurus,” was identified in India.^4^, ^5^ BA.4.6, another sublineage emerged almost simultaneously in many countries. Many lineages and sublineages of this rapidly transmitting and highly severe superinfectious Omicron are listed in the “VOC lineages under monitoring” (VOC‐LUM) category by the WHO which are being monitored.^2^ As the seemingly more infectious new strains than earlier ones emerge, coexisting with the virus seems to be the “new normal” at least in the near future. That the virus will keep evolving is of concern that may worry the healthcare infrastructure. Virus mutation is usual, an evolution measure to defy immunity. With the virus living longer in immunosuppressed individuals, new unidentified lineages and sublineages may emerge in all likelihood.^6^ Plenty of the variants expectedly may eventually fizzle.

As the latest Omicron sublineage persists, health officials and researchers keep eye as to how the future unfolds. Experts are closely tracking the novel sublineages including the recent XBB.1.16 responsible for the rising COVID‐19 cases, especially in India (https://www.hindustantimes.com/lifestyle/health/all-about-xbb-1-16-new-omicron-variant-behind-india-s-covid-19-spike-from-symptoms-to-risk-factors-101680160372702.html). XBB is a hybrid of two globally circulating Omicron subvariants. XBB.1.16 is dangerous due to its high contagiousness and rapid spread. This highly transmissible and highly infectious hybrid lineage is nicknamed Arcturus. Although whether it eventually spreads to worrisome levels is not clear, however, it has raised concerns. The earlier dominant variants are being replaced rapidly with this lineage mutating in the non‐spike region (ORF9b mutations) which may facilitate efficient immunity evasion. XBB.1.16 subvariant has been detected in India, the United States, Australia and the UK. The infection rate of the subvariant is still less attributable to the acquired (vaccination drive) and innate immunity (from prior infection). Acquired hybrid immunity among the human population mostly exposed to Omicron variant could be possible.^7^ Whether new sublineages could outcompete the previous ones is important to decipher; it is too early to comprehend. In the scenario of fresh rise in cases, it could potentially lead to yet another all‐too‐familiar cycle. As the newer lineages and sublineages still mutate, it is hard to hypothesize the course it may take becoming the next dominant variant for another wave, if any.

Omicron sublineage BA.4.6 (designated as V‐22SEP‐01) spread in the UK after being first identified from a specimen on May 12, 2022 there. BA.4.6 has mutation in an antigenically significant site (S: R346T) and has an apparent growth advantage over BA.5. Pseudoviral neutralization assay by the University of Oxford reported that BA.4.6 had a twofold reduced titer as compared with BA.4 and BA.5 neutralization by the sera from triple‐dose recipients of Pfizer's BNT162b2 vaccine.^8^ This worries as it suggests that COVID vaccines may be less effective against BA.4.6. With new Omicron sublineages hitting primarily the US and the UK population, the possible transboundary transmission is high as international travel restrictions are relaxed. Owing to the fact that countries like China, where there had been the depth (infection severity) and spread (rapid transmission) concerns about the lethal pandemic, lift international travel restrictions opening up their air space worries the world.

## 2 METHODS

Tracking of SARS‐CoV‐2 variants worldwide during the pandemic progressed significantly with rapid and near real‐time broadcasting of genomic data from agencies and institutions. Platforms like COVID‐19 data portal,^9^ Nextstrain,^10^ and tools like outbreak.info^11^ systematically document the genomic diversity and distribution of SARS‐CoV‐2 lineages. Daily sample data of SARS‐CoV‐2 variants in circulation relevant to the epidemiological situation during the study period was collected from the GISAID database.^12^ Statistical tools were used to analyze the data, and the relative growth advantages of the variants to the frequency of the daily data samples of variants were estimated using a logistic model. This model estimated the logistic growth rate and quantified the fitness advantage of the global transmission of variants. Please refer to the GitHub repository (https://github.com/cevo-public/Quantification-of-the-spread-of-a-SARS-CoV-2-variant) for additional details on the methodology and the implementation of the logistic model.

The reproductive number (*R*_e_), a key indicator to describe how efficiently a pathogen spreads in a given population at a given time, was also estimated using the EpiEstim R package. It estimated the *R*_e_ by modeling the disease transmission with Poisson process. An individual who got infected at an earlier time “*t* − *s*,” considering a specific time point (denoted as “*t*”), becomes the source of a new infection. The rate at which these new infections occur is determined by the product of the time‐varying effective reproduction number, *R*_e_(*t*), and the infectivity weight, “*w*_*s*_” which signifies the infectivity level *s* days after the initial infection. The likelihood associated with the occurrence of new cases, denoted as “*I*_t_” at the time point “*t*” can be expressed as:

P(It|IO,…,It−1,Re(t))=(R(t)eΔt)Ite−Re(t)ΔtI!t,

where, $\Delta_{t}=\sum_{s=1}^{t}I_{t‐s}w_{s}$ represents the cumulative weighted sum of past incidences up to the time *t*, as defined earlier.

## 3 RESULTS

**Figure 1:**
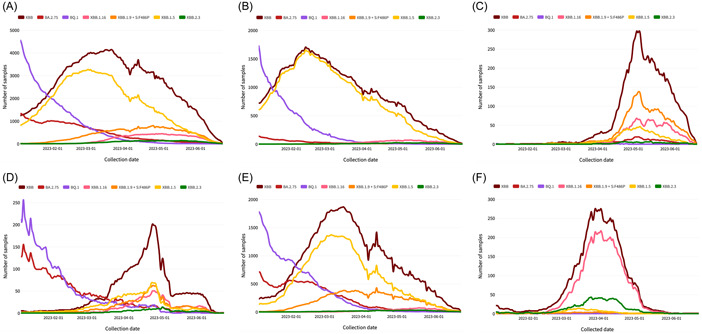
Average daily cases during January–June 2023; (A) World; (B) USA; (C) China; (D) Japan; (E) Europe; (F) India.

Figure 1 depicts the collected average daily data samples (more than 1 million) of the circulating SARS‐CoV‐2 variants during January and June 2023. BQ.1, along with BA.2.75, was the most prevalent SARS‐CoV‐2 sublineage worldwide in early 2023 (Figure 1A), in the United States (Figure 1B), Europe (Figure 1E), and Japan (Figure 1D). XBB variant strain dominated (about 57.63% among sequenced samples) worldwide between March and June, followed by XBB.1.5 (37.95%), although several other variants had a significant global impact including XBB.1.9 with an F486P spike protein mutation identified in China (Figure 1C) and Europe (Figure 1E), and XBB.1.16 and XBB.2.3 in India (Figure 1F).

### 3.1 Relative growth advantages

**Figure 2:**
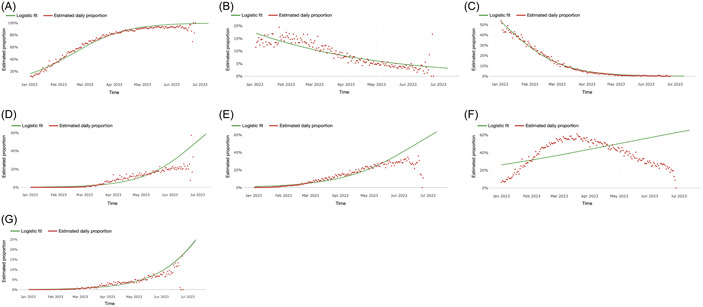
Estimated relative weekly growth advantage of global variants; (A) XBB; (B) BA.2.75; (C) BQ.1; (D) XBB.1.16; (E) XBB.1.9+S:F486P; (F) XBB.1.5; (G) XBB.2.3.

A logistic model to the frequency of the daily samples of variants was applied to estimate the logistic growth rate and quantify the fitness advantage of the global transmission of the variants. The estimated relative growth advantage reflected the advantage over the other co‐circulating variants. Thus, the focal variant advantage may decrease as a new variant spreads. The three factors that may alter the intrinsic growth rate are intrinsic transmission advantage, immune evasion, and prolonged infectious period.^13^ The estimate *e* ^(*a*)^‐1, where *a* is estimated logistic weekly growth rate taking into account all the three factors (Figure 2), XBB had an estimated 28% relative weekly growth advantage compared with other globally‐circulating variants (Figure 2A), followed by 25% in XBB.1.16 (Figure 2D), 22% in XBB.2.3 (Figure 2G), and 19% of XBB.1.9+S:F486P (Figure 2E).

### 3.2 Reproductive number (*R_e_*)

**Figure 3:**
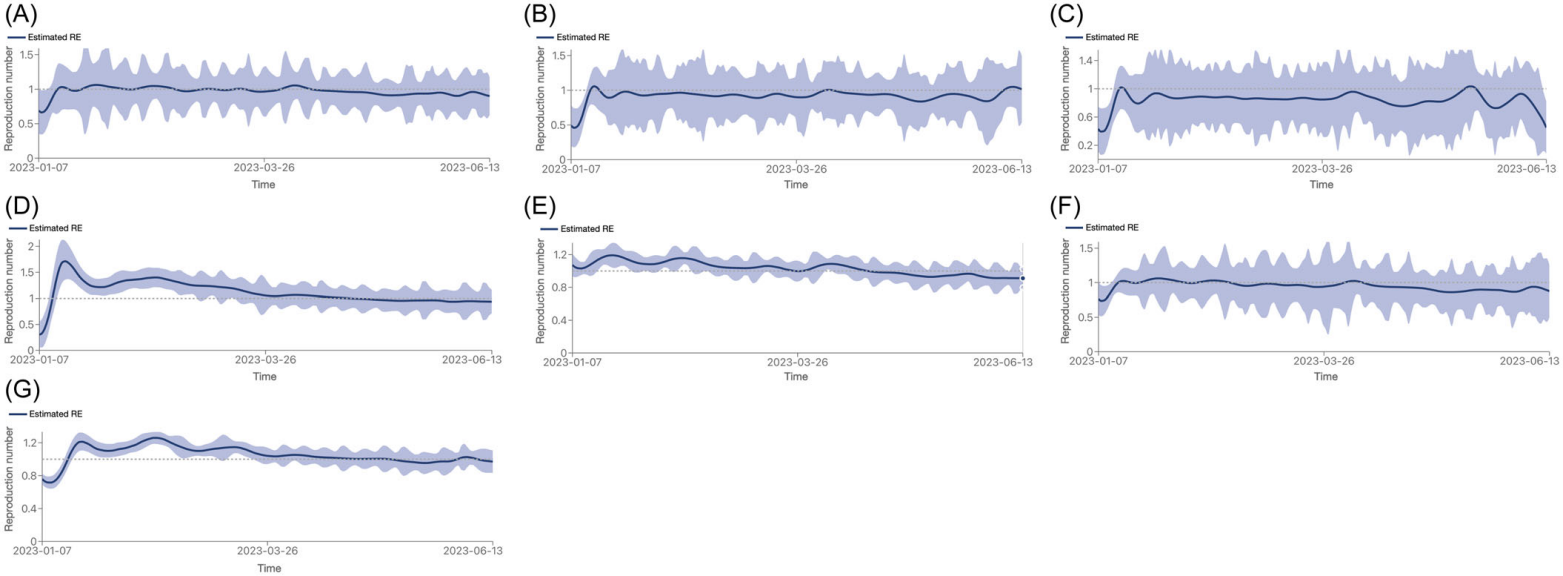
Estimated reproduction number (*R*_*e*_) per variant on a global scale. (A) XBB; (B) BA.2.75; (C) BQ.1; (D) XBB.1.16; (E) XBB.1.9+S:F486P; (F) XBB.1.5; (G) XBB.2.3.

Following the method of Huisman and coworkers,^14^ reproductive number of the variants on global scale (*R*_*e*_) was estimated (Figure 3). The variant was increasingly infecting more people if *R*_*e*_ was greater than one, the number of infected cases was declining but if it was less than one. For instance, the *R*_*e*_ for XBB that was dominant within few weeks was estimated to be 1.06 (0.62–1.50; 95% highest posterior density interval [HPD]) based on whole genome sequencing data during January 24–27, 2023. A detailed country‐specific comparison among the value estimates of *R*_*e*_ per variant during the study is summarized in Table 1.

**Table 1:** The estimated reproduction number (*R*_*e*_) values during the study.

Geographical region	Period	Reproduction number (*R*_*e*_)	
		Variant	*R*_*e*_ values^a^
World	5 January–14 June 2023	XBB	0.96 (0.76–1.17)
		BA.2.75	0.92 (0.67–1.17)
		BQ.1	1.02 (0.50–1.54)
		XBB.1.16	0.97 (0.72–1.21)
		XBB.2.3	1.02 (0.92–1.11)
		XBB.1.9+S:F486P	0.96 (0.84–1.08)
		XBB.1.5	0.91 (0.71–1.12)
USA	5 January–14 June 2023	XBB	0.66 (0.00–7.54)
		BA.2.75	0.99 (0.00–11.33)
		BQ.1	1.00 (0.00–11.42)
		XBB.1.16	0.99 (0.00–1.34)
		XBB.2.3	1.00 (0.00–11.40)
		XBB.1.9+S:F486P	0.99 (0.00–11.34)
		XBB.1.5	0.92 (0.00–10.58)
China	5 January–14 June 2023	XBB	0.89 (0.48–1.31)
		BA.2.75	0.42 (0.00–0.95)
		BQ.1	0.65 (0.00–7.41)
		XBB.1.16	0.78 (0.40–1.16)
		XBB.2.3	1.12 (0.91–1.35)
		XBB.1.9+S:F486P	0.95 (0.78–1.11)
		XBB.1.5	0.74 (0.52–0.96)
Japan	5 January–14 June 2023	XBB	0.98 (0.00–11.21)
		BA.2.75	1.00 (0.00–11.42)
		BQ.1	1.00 (0.00–11.44)
		XBB.1.16	0.99 (0.00–11.36)
		XBB.2.3	1.00 (0.00–11.42)
		XBB.1.9+S:F486P	0.99 (0.00–11.38)
		XBB.1.5	1.00 (0.00–11.40)
Europe	5 January–14 June 2023	XBB	0.73 (0.56–0.90)
		BA.2.75	0.85 (0.66–1.04)
		BQ.1	0.04 (0.00–0.23)
		XBB.1.16	0.70 (0.57–0.83)
		XBB.2.3	0.84 (0.78–0.91)
		XBB.1.9+S:F486P	0.80 (0.73–0.88)
		XBB.1.5	0.78 (0.59–0.98)
India	5 January–29 May	XBB	0.68 (0.58–0.78)
		BA.2.75	0.39 (0.00–4.46)
		BQ.1	1.00 (0.00–11.45)
		XBB.1.16	0.42 (0.24–0.61)
		XBB.2.3	0.00 (0.00–0.03)
		XBB.1.9+S:F486P	1.26 (1.03–1.51)
		XBB.1.5	0.85 (0.42–1.28)

^a^ *R*_*e*_ at 95% highest posterior density interval.

### 3.3 Vaccine effectiveness

Omicron sublineages (BA.1‐5, BA.2.75, and BA.4.6) and XBB, XBB.1.5 and XBB.1.16 have emerged globally. BA.4.6 allegedly shows higher resistance to a mAbs class possibly due to mutating R346T/S/I.^15^ Reports suggest that other Omicron subvariants including BA.5 could also be resistant to mAbs, and even to cilgavimab/tixagevimab. Approved combination of cilgavimab and tixagevimab completely lost the neutralization effect against BA.4.6.^15^ The mAb Bebtelovimab was potent against BA.4.6 sublineage while Evusheld was not.^16^ Study showed that BA.4.6 could escape vaccine immunity better than BA.5, demonstrating an average 2.4–2.6‐fold decrease in antibody neutralization. It suggested that bebtelovimab is the only therapeutic mAb that has retained potency against all these circulating variants.

BA.4.6 is a descendant of the BA.4 Omicron variant. Although BA.4.6 is similar to BA.4 in numerous ways, it carries a mutated s‐protein (responsible for the entry of the cell). This mutation (R346T) has also been reported in variants associated with immunity‐evasion helping the virus escape antibodies acquired from vaccination and prior infection(s). How BA.4.6 has emerged is not yet entirely clear, but possibly it is a recombinant variant, possibly occurred when two SARS‐CoV‐2 variants simultaneously infected the same (immunucompromised?) host.^17^ The WHO has warned of “XE,” a recombinant virus (combination of BA.1 and BA.2).^18^ BA.4.6, a BA.4 sublineage, shares mutations with it but has an acquired mutation in a potentially significant site for antigenicity spike: R346T. Due to the increasing similarities across BA sublineages, assigning lineages to the sequences with lower genome coverage is difficult. Sublineage BA.2.75 has been reported in samples from India, United States, Singapore, Australia, Japan, Canada, and the UK. The infectivity of BA.2.75 grew between July and August, 2022. BA.4.6 reportedly has 6.55% relative fitness advantage over BA.5, considerably smaller than the advantage of BA.5 over BA.2.

Chinese researchers opined that the Omicron sublineage BA.4.6 exhibited extensive growth advantages as compared with BA.4 and BA.5. Vaccines and boosters offer the best protection against severity and possible death. Immunity that could help decelerate the emergence of novel variants and their spread depends on vaccination cover. Pfizer and Moderna vaccines are approved for emergency use against Omicron variant in the United States and the UK. As the existing inactivated vaccines from wild‐type strain seem inefficient even beyond the booster dose, scoping for effective novel vaccines against Omicron variants is crucial. Six clinical trials of inactivated and nine of mRNA‐based Omicron variant vaccine candidates (15 clinical trials in all) were being carried out as by mid‐2022.^19^ As the emergence of SARS‐CoV‐2 variants is not well understood, developing Omicron variants specific vaccines is challenging. It is suggested that governments should plan for routine annual COVID boosters similar to seasonal flu and other diseases until herd immunity is attained. COVID‐19 surges are likely to continue as they develop mutations, and until the next‐gen vaccines roll them out equitably worldwide.^20^ 67.5% of US population is vaccinated (https://covid.cdc.gov/covid-data-tracker/#vaccinations_vacc-people-onedose-pop-5yr) and 48.5% of them received booster shot, as per CDC. It is important to note that the booster shot was administrated long ago. Keeping in mind the continuously emerging novel Omicron subvariants, the provision of extra vaccine shots to the population is recommended.

Currently, the SARS‐CoV‐2 descendants BQ.1 and BQ.1.1, as well as recombinant XBB, are gaining dominance worldwide. BQ.1.1 has three more substitutions (R346T, K444T, N460K) in its RBD region than BA.5. The XBB has nine more changes (R346T, G339H, L368I, G446S, N460K, V445P, F490S, F486S, and the wild‐type amino acid at position 493) in its RBD than BA.2.^21^ Mutated XBB could evade immunity while its infective ability reduced, and XBB.1.5 evolved with better infectivity while additionally dodging immunity protection thus spreading easily. These variants attached to the upper airway cells to infect without really traveling deep down to the lungs. The subvariants BQ and XBB could entirely resist the prevailing mAb treatments.^22^ Further, the levels of neutralizing antibody were 12–26 times lower against novel Omicron subvariants including XBB compared with original SARS‐CoV‐2 strain in bivalent booster recipients.^22^ As compared with earlier strains, the antibodies of the previously infected and vaccinated patients were 49–63 times less likely to neutralize XBB.1 subvariant.^22^ Antiviral therapies like nirmatrelvir/ritonavir and remdesivir are still resorted to as mAb treatments against the evolving Omicron subvariants seem less effective.

Due to the unique mutations in the spike and RBD of Omicron and its lineages, the efficacy of the current vaccines and mAb therapies is debatable.^23^ Recently, a bivalent vaccine consisting of s‐proteins of the original SARS‐CoV‐2, and BA.4‐5 (Omicron) manufactured by Moderna and Pfizer‐BioNTech is approved by the USFDA.^24^ The efficacy of mAb therapies against Omicron variants are listed in Table 2.^28^ To block the cell‐virus fusion region (COVID19‐SF5), an IgG antibody was synthesized that cross‐reacted at six cell‐adhesion spike protein sites.^29^ Such therapeutic antibodies might restrict viral replication, minimize mutations, and obstruct infections. Increasing infection by the spreading variants could adversely affect nations and economies. In light of it, existing vaccines could be reevaluated and vaccination strategies against the variants could be improved.

**Table 2:** Efficacy of mAb therapies against Omicron variants.

mAb	Clinical manifestation	Reference
Bebtelovimab	Effective against Omicron BA.1‐2, BA.4‐5	[25, 26, 27]
Casirivimab	Effective against Omicron	[25, 26, 27]
Tixagevimab	Moderately effective against Omicron	[25, 26, 27]
Imdevimab	Moderately effective against Omicron	[25, 26, 27]
Cilgavimab	Moderately effective against Omicron	[25, 26, 27]
Tixagevimab+ Cilgavimab	Moderately effective against Omicron BA.1‐2, BA.4‐5	[25, 26, 27]
Bebtelovimab+ Etesevimab	Effective against Omicron BA4‐5	[25]
Imdevimab+casirivimab, tixagevimab+cilgavimab, sotrovimab, bebtelovimab	May not be effective against BQ.1.1/XBB	[21]

### 3.4 Technical validation

Multiple methodologies and guidelines were used to evaluate the quality of the study. Supporting Information: Table S1 describes the STROBE Guidelines (Strengthening the Reporting of Observational Studies in Epidemiology),^30^ an influential initiative aimed at enhancing the transparency and quality of reporting for observational research studies. STROBE provides comprehensive guidelines and checklist offering researchers a structured framework to transparently present the finding in epidemiological studies. STROBE supports clear statement of study design, methods, results and conclusions by emphasizing on the accurate and thorough reporting. It plays a crucial role in improving the overall reliability and interpretability of observational research, enabling researchers, practitioners, and policymakers to make informed decisions based on sound evidence. Responses against each STROBE guideline clause are provided.

Critical Appraisal Skills Program (CASP) checklist was applied (Supporting Information: Table S2) to evaluate the methodological quality of the study. CASP (https://casp-uk.net/) provides practical tools, guidelines, and educational resources to empower healthcare professionals, researchers, and students to assess the quality, validity, and relevance of research articles, systematic reviews, and other studies. By promoting rigorous evaluation techniques, CASP enhances the ability to make informed decisions, interpret findings, and apply evidence‐based practices. The program's emphasis on transparent, comprehensive appraisal contributes to the robustness and credibility of evidence‐driven decision‐making and research endeavors. The details related to the same against each question are discussed below.

## 4 DISCUSSION

This paper highlights the significant challenges posed by the emerging Omicron's subvariants vis‐a‐vis the vaccine efficacy and current preparedness. Vaccination continues to offer good protection against severe diseases and is still the best weapon against COVID‐19 pandemic. The Medicines and Healthcare products Regulatory Agency (MHRA) approved the Spikevax bivalent COVID‐19 vaccine by Moderna that targets two coronavirus (CoV) variants for adult booster dose.^31^ It met the UK regulator's standards of safety, quality and efficacy as per their independent expert scientific advisory body. This bivalent vaccine triggers a strong immune response against both the original 2020 strain and Omicron BA.1. It also generated a good immune response against other (BA.4 and BA.5) Omicron subvariants without serious safety concerns.^31^ There is an urgent need to develop next‐gen multivalent vaccines in similar lines for long‐lasting wide‐spectrum protection. As per a recent animal trial, the respiratory mucosal delivery of Adjuvant‐vectored multivalent vaccine may be an effective strategy for the next‐gen COVID‐19 vaccine against the currently circulating and future SARS‐CoV‐2 VOCs.^32^ Imbalances at environment and individual human levels coupled with the unplanned nonecofriendly economic activities seem to have reached the brink wherein multiple incidences of critical global health concerns by the evolving and novel etiological agents may be triggered. As also opinionated by various global health agencies including the WHO, it is certainly a public health challenge in the “new normal.” As is understood, Omicron subvariants are extremely transmissible and can even evade natural and vaccine‐induced immunity. Global in vivo, in vitro, or in silico scientific advancements in biological sciences at especially the molecular level has empowered conceptualizing and strategizing the development of next‐gen vaccine in the ongoing scenario to effectively counter COVID‐19 pandemic. This could effectively work against the virus and many of its evolving variants and subvariants. Such vaccines should be effective not only regionally but also globally. Any such success in the ever‐encompassing wide‐spectrum COVID vaccine could pave the way for many other such existing as well as future maladies of global concern.

In addition, by leveraging advanced genomic sequencing and statistical analysis, researchers could track the emergence and spread of the variants, limiting the impact of fresh COVID‐19 waves with: (i) data interpretation, that is, identifying patterns, trends, and relationships within data to develop an understanding of the characteristics and behavior of different variants; (ii) comparisons and associations, that is, comparing COVID‐19 variants with various associated factors like disease severity, transmission rates, vaccine efficacy and demographic variables; (iii) predictive modeling, that is, developing predictive models to predict future behavior and prevalence of COVID‐19 variants; and (iv) risk assessment and decision making, that is, providing quantitative measures to assess the associated risk with different COVID‐19 variants. By combining the variant's data with the epidemiological and clinical information, statistical methods could estimate the likelihood of the variant‐related outcomes like disease severity, increased transmissibility, breakthrough infections, or even the effect of vaccine avoidance. Such risk assessments and predictive modeling would empower public health authorities, policymakers, and healthcare providers to take informed decision and implement appropriate measures to mitigate the spread and impact of the circulating variants.

The pricing of COVID‐19 vaccine varies widely among countries and companies, varying between high‐income to middle‐income to low‐income countries. Some pharma companies had offered their COVID‐19 vaccine on not‐for‐profit bases across countries. The financial outlay amounted to US$2.018 billion at the global level, equivalent to US$1.66 per dose and US$3.70 per person with double‐dose vaccination (accounting also for the wastage) (https://www.who.int/publications/m/item/costs-of-delivering-covid-19-vaccine-in-92-amc-countries). Studies suggest that COVID‐19 vaccination was a cost‐effective (cost‐saving) intervention to mitigate the viral transmission irrespective of the circumstances or situation.^33^ Studies reported that the vaccine efficacy values were 65%–75%. It is important to plan for vaccination scale‐up, like determining the required quantity, coverage rate, target population, vaccination strategy, and so forth, for vaccine costing. Direct medical costs are usually hospitalization, treatment, and the vaccination costs.^34^, ^35^, ^36^, ^37^, ^38^ Studies also consider diagnostics/testing costs in the direct medical costs.^33^ Vaccine wastage, human resource, logistics, contact trace, quarantine, and vaccination campaign are the nonmedical costs.^38^ The indirect costs also could include the economic loss due to the COVID‐19 pandemic.^36^, ^38^ Vaccine efficacy, vaccination coverage and priority of administration in certain groups are the primary factors in vaccine cost‐effectiveness. Vaccination might be of no use if administered to wrong population.^37^, ^38^ In the face of a possible next pandemic, the discussion may help the policymakers worldwide.

India has efficiently mass vaccinated its majority population. The vaccination rate was lower in rural India as compared with urban.^39^ Regions with high population burden per health center likely had lower vaccination rates. The vaccination was reportedly lower among the pregnant and the lactating. As per a recent cross‐sectional survey in urban resettlement colony, slum, and village cluster in the Indian capital Delhi, more than four in every 10 adults did not receive the booster dose despite higher (~95%) rate of double dose vaccination.^40^ Nearly 70% Indians were vaccinated and nearly 10% were booster‐dose vaccinated (https://vaccinate-india.in/dashboard). Higher death rates among people with chronic diseases (like diabetes and kidney ailments) and the old were observed when the Delta and Omicron variants dominated in a study conducted in 110 countries.^41^ The death rate was relatively low among the fully vaccinated. Comparing the time periods, death was 3.45‐fold high during Delta as compared with Alpha variant.^42^ Omicron was observed to be less lethal than Delta.^43^ Thus, COVID‐19‐associated fatality was strongly linked with vaccination coverage, prevailing individual health burden and the governmental response. Public health program is suggested to enhance its focus on improved access to vaccination services particularly in the underserved areas to support the vulnerable groups.

## 5 LIMITATIONS OF THE STUDY

Each study has its own inherent limitation, and this reported study is no exception to it. The study is based on genomic data from GISAID. It may not represent the entire population as it is based on only the samples that were sequenced and shared by researchers and public health agencies. Thus, the results could not be generalized to the entire population. Further, the study does not explicitly inform on the characteristics of the study participants, missing data, or potential constraints that limit the ability to control the confounding or to generalize the results to other populations. Also, the study covers the period from January to June 2023 only, not accounting for current epidemiology or the emerging novel variants. The study may not capture the full impact of the Omicron subvariants or other emerging variants emerging in the future.

## 6 CONCLUSION

This work comprehensively analyses the prevalence and impact of SARS‐CoV‐2 variants and highlights the importance of effective statistical analyses in tracking emergence and spread. It also provides leads to investigate potential next‐gen vaccines that effectively combats the continuously emerging Omicron subvariants.^44^ Considering the potential limitations of the study is important, like the brevity and completeness of the data from the GISAID database that may not represent global population, and the limited timeframe of the analysis that may lack capturing the full extent of the emergence and spread of variants. The recommendations for potential future directions for effectively strategize for a next‐gen vaccine are as follows:

1. *Continual monitoring and sequencing of variants*: New variants of concern could lead to the emergence of fresh COVID‐19 pandemic wave. Thus, it is crucial to closely monitor and sequence new variants to design next‐gen vaccine and stay safe. Recommendations may focus on boosting global alliance and investment in real‐time genome surveillance to detect and track the emerging variants.
2. *Targeted vaccine development*: An effective strategy to prevent infectious diseases is developing targeted vaccines. Focus on developing (variants or subvariants) targeted vaccines for improved vaccine efficacy and reduced risk of breakthrough infections could be recommended.
3. *Rapid vaccine development and distribution*: The study emphasizes global alliance for rapid vaccine development and its effective distribution to combat the emerging variants. Improved and fool‐proof vaccine development strategies, better distribution, easy scaled‐up vaccine production, and ensuring equitable access to vaccines for all are recommended.
4. *Testing the safety and efficacy of next‐gen vaccines*: Next‐gen vaccine could potentially combat the continuously emerging Omicron subvariants. Conducting rigorous testing on the safety and efficacy of next‐gen vaccine before approval for use is, therefore, needed.
5. *Developing predictive models*: The use of statistics effectively is crucial in the face of emerging novel variants of concern to limit any COVID‐19 wave in the future. A focus on developing predictive models to forecast the emergence and spread of new variants to facilitate public health policy, preparedness, and implementation is suggested.

## AUTHOR CONTRIBUTIONS

**Ranjan K. Mohapatra**: Conceptualization; supervision; writing—original draft. **Snehasish Mishra**: Project administration; writing—review and editing. **Venkataramana Kandi**: Validation; writing—original draft. **Francesco Branda**: Data curation; formal analysis; writing—original draft. **Azaj Ansari**: Writing—original draft. **Ali A. Rabaan**: Validation; writing—original draft. **Kudrat‐E‐Zahan**: Project administration; writing—review and editing.

## CONFLICT OF INTEREST STATEMENT

The authors declare no conflict of interest.

## TRANSPARENCY STATEMENT

The lead author Ranjan K. Mohapatra, Kudrat‐E‐Zahan affirms that this manuscript is an honest, accurate, and transparent account of the study being reported; that no important aspects of the study have been omitted; and that any discrepancies from the study as planned (and, if relevant, registered) have been explained.

## Supporting information

Supporting information.

## Data Availability

All the data are not publicly available due to privacy or ethical restrictions. Such data supporting the findings of this study are available from the corresponding authors on a reasonable request.
