# Supplementary material for: Analyzing the emerging patterns of SARS‐CoV‐2 Omicron subvariants for the development of next‐gen vaccine: An observational study
Source: Health Sci Rep. 2023 Oct 18;6(10):e1596. doi: 10.1002/hsr2.1596 (PMC10584996; doi:10.1002/hsr2.1596)
Supplement: Supplementary file 1 — Supporting information. [file HSR2-6-e1596-s001.doc]

***Supplementary Information***

**Analysing the Emerging Patterns of SARS-CoV-2 Omicron Subvariants for the Development of Next-Gen Vaccine: An Observational study**

Ranjan K. Mohapatra^1^,*^,#^, Snehasish Mishra^2,#^, Venkataramana Kandi^3^, Francesco Branda^4^, Azaj Ansari^5^, Ali A. Rabaan^6,7,8^, Md. Kudrat-E-Zahan^9^,*

^1^Department of Chemistry, Government College of Engineering, Keonjhar – 758 002, Odisha, India

^2^School of Biotechnology, Campus-11, KIIT Deemed-to-be-University, Bhubaneswar, Odisha – 751 024, India

^3^Department of Microbiology, Prathima Institute of Medical Sciences, Karimnagar – 505 417, Telangana, India

^4^Department of Computer Science, Modeling, Electronics and Systems Engineering (DIMES), University of Calabria, Rende, Italy

^5^Department of Chemistry, Central University of Haryana, Mahendergarh – 123 031, Haryana, India

^6^Molecular Diagnostic Laboratory, Johns Hopkins Aramco Healthcare, Dhahran 31311, Saudi Arabia

^7^College of Medicine, Alfaisal University, Riyadh 11533, Saudi Arabia

^8^Department of Public Health and Nutrition, The University of Haripur, Haripur 22610, Pakistan

^9^Department of Chemistry, Rajshahi University, Rajshahi-6205, Bangladesh

#both the authors have same contribution and are treated as first authors.

***Correspondence:**

**Dr. R. K. Mohapatra**, PhD

Phone: +91- 7008666708

Email: ranjank_mohapatra@yahoo.com; https://orcid.org/0000-0001-7623-3343

**Prof. Dr. Kudrat-E-Zahan**, PhD

Rajshahi University, Rajshahi-6205, Bangladesh

Email: [kudrat.chem@ru.ac.bd](mailto:kudrat.chem@ru.ac.bd); https://orcid.org/0000-0001-8159-5293

**Table S1.** The STROBE statement [32]

|  | ***Item*** | ***STROBE recommendation*** | ***Response*** |
| --- | --- | --- | --- |
| ***TITLE*** ***and ABSTRACT*** | 1 | (a) Indicate the study's design with a commonly used term in the title or the abstract | The work clearly indicates in the title the type of study conducted, while the abstract is organised into four main sections: “Background and Aims”, “Methods”, “Results”, and  “Conclusion” to provide an informative and balanced summary of what was done and what was found. |
|  |  | (b) Provide in the abstract an informative and balanced summary of what was done and what was found |  |
| ***INTRODUCTION*** | | |  |
| ***Background/***  ***rationale*** | 2 | Explain the scientific background and rationale for the investigation being reported | The authors state that understanding the prevalence and impact of SARS-CoV-2 variants has assumed paramount importance, and effective statistical analysis is crucial in tracking the emergence and spread of these variants. The study aims to estimate the transmission fitness advantage and the effective reproductive number (Re) of the SARS-CoV-2 sub-lineages that are relevant to the epidemiological situation in the period under analysis through time based on data from GISAID. The authors also investigate the potential of next-gen vaccines to combat continuously emerging Omicron subvariants.  Overall, the scientific background and rationale for the investigation being reported is to provide valuable insights into the development of next-gen vaccines and the tracking of emerging variants, which can inform public health policies and strategies to combat the COVID-19 pandemic. |
| ***Objectives*** | 3 | State specific objectives, including any prespecified hypotheses | The study aims to estimate the transmission fitness advantage and the effective reproductive number (R_e_) of the SARS-CoV-2 sub-lineages that are relevant to the epidemiological situation in the period under analysis through time based on data from GISAID. The authors also investigate the potential of next-gen vaccines to combat continuously emerging Omicron subvariants.  The authors highlight that effective statistical analysis can provide valuable insights into the prevalence and impact of SARS-CoV-2 variants, and that next-gen vaccines may be effective in combating emerging Omicron subvariants. |
| ***METHODS*** | | |  |
| ***Study design*** | 4 | Present key elements of study design early in the paper | The work provides a clear and concise overview of the study design in the abstract section. The study design includes estimating the transmission fitness advantage and the effective reproductive number (Re) of the SARS-CoV-2 sub-lineages that are relevant to the epidemiological situation in the period under analysis through time based on data from GISAID. The study covers the period from January to June 2023, encompassing a wide range of sequenced samples, and identifies the dominance of the XBB variant strain, accounting for approximately 57.63% of cases, during this timeframe.  Overall, the key elements of study design, including the objectives, methods, and results, are presented early in the paper to provide readers with a clear understanding of the study's scope and findings. |
| ***Setting*** | 5 | Describe the setting, locations, and relevant dates, including periods of recruitment, exposure, follow-up, and data collection | The study does not provide specific information on the setting or locations of the study. However, the work covers the period from January to June 2023, and the data used for the analysis were obtained from GISAID.  The study does not involve recruitment, exposure, or follow-up of participants, as it is a statistical analysis of SARS-CoV-2 sub-lineages based on data from GISAID. |
|  |  |  |  |
| ***Participants*** | 6 | (a) *Cohort study*—give the eligibility criteria, and the sources and methods of selection of participants. Describe methods of follow-up  *Case-control study*—give the eligibility criteria, and the sources and methods of case ascertainment and control selection. Give the rationale for the choice of cases and controls  *Cross-sectional study*—give the eligibility criteria, and the sources and methods of selection of participants | The study does not involve recruitment, exposure, or follow-up of participants, as it is a statistical analysis of SARS-CoV-2 sub-lineages based on data from GISAID. |
|  |  | (b) *Cohort study*—for matched studies, give matching criteria and number of exposed and unexposed  *Case-control study*—for matched studies, give matching criteria and the number of controls per case |  |
| ***Variables*** | 7 | Clearly define all outcomes, exposures, predictors, potential confounders, and effect modifiers. Give diagnostic criteria, if applicable | The study focuses on the transmission fitness advantage and the effective reproductive number (R_e_) of the SARS-CoV-2 sub-lineages based on data from GISAID and identifies the dominance of the XBB variant strain during the period from January to June 2023. The study does not mention any specific exposures, potential confounders, or effect modifiers. |
| ***Data sources/***  ***measur ement*** | 8 | For each variable of interest give sources of data and details of methods of assessment (measurement). Describe comparability of assessment methods if there is more than one group | The data source for the study is GISAID, and the methods of assessment involve statistical analysis of the data for the outcome of interest and sequencing and analysis of the viral genomes for the predictors of interest. |
| ***Bias*** | 9 | Describe any efforts to address potential sources of bias | The study uses various statistical methods to estimate the relative growth advantages of the variants and the effective reproductive number (R_e_) of the SARS-CoV-2 sub-lineages, which can help to minimize potential sources of bias in the analysis. |
| ***Study size*** | 10 | Explain how the study size was arrived at | The study covers the period from January to June 2023 and encompasses a wide range of sequenced samples. The sample size is more than 1 million, i.e., all relevant SARS-CoV-2 sub-lineages that were sequenced and available in the GISAID database during the period under analysis. |
| ***Quantitative variables*** | 11 | Explain how quantitative variables were handled in the analyses. If applicable, describe which groupings were chosen, and why | The study used several statistical methods to analyze quantitative variables, such as growth rates. In addition, the authors chose specific groupings of the quantitative variables based on relevant factors such as disease severity and transmission rates. |
| ***Statistical***  ***methods*** | 12 | (a) Describe all statistical methods, including those used to control for confounding | The study describes various statistical methods to estimate the relative growth advantages of the variants and the effective reproductive number (R_e_) of the SARS-CoV-2 sub-lineages. However, it includes all relevant SARS-CoV-2 sub-lineages that were sequenced and available in the GISAID database during the period under analysis. |
|  |  | (b) Describe any methods used to examine subgroups and interactions |  |
|  |  | (c) Explain how missing data were addressed |  |
|  |  | (d) *Cohort study*—if applicable, explain how loss to follow-up was addressed  *Case-control study*—if applicable, explain how matching of cases and controls was addressed  *Cross-sectional study*—if applicable, describe analytical methods taking account of sampling strategy |  |
|  |  | (e) Describe any sensitivity analyses |  |
| ***RESULTS*** |  |  |  |
| ***Participants*** | 13 | (a) Report the numbers of individuals at each stage of the study—eg, numbers potentially eligible, examined for eligibility, confirmed eligible, included in the study, completing follow-up, and analysed | N/A* |
|  |  | (b) Give reasons for non-participation at each stage |  |
|  |  | | | (c) Consider use of a flow diagram | | --- | | | --- | --- | |  |
| ***Descriptive data***  ***on exposures and potential confounders*** | 14 | (a) Give characteristics of study participants (eg, demographic, clinical, social) and information on exposures and potential confounders | N/A* |
|  |  | (b) Indicate the number of participants with missing data for each variable of interest |  |
|  |  | (c) *Cohort study*—summarise follow-up time (eg, average and total amount) |  |
| ***Outcome data*** | 15 | *Cohort study*—report numbers of outcome events or summary measures over time | N/A* |
|  |  | *Case-control study*—report numbers in each exposure category, or summary measures of exposure |  |
|  |  | *Cross-sectional study*—report numbers of outcome events or summary measures |  |
| ***Main results*** | 16 | (a) Give unadjusted estimates and, if applicable, confounder-adjusted estimates and their precision (eg, 95% confidence interval). Make clear which confounders were adjusted for and why they were included | N/A* |
|  |  | (b) Report category boundaries when continuous variables were categorised |  |
|  |  | (c) If relevant, consider translating estimates of relative risk into absolute risk for a meaningful time period |  |
| ***Other analyses*** | 17 | Report other analyses done—eg, analyses of subgroups and interactions, and sensitivity analyses | The study provides a comprehensive analysis of the emerging Omicron subvariants and their potential impact on vaccine efficacy and preparedness. While it does not provide explicit information on subgroup analyses, interactions, or sensitivity analyses, it provides valuable insights and strategic approaches to guide vaccine formulation and preparedness. |
| ***DISCUSSION*** |  |  |  |
| ***Key results*** | 18 | Summarise key results with reference to study objectives | The study aimed to analyze the emerging Omicron subvariants and their potential impact on next-gen vaccine strategies. It estimated the transmission fitness advantage and the effective reproductive number (R_e_) of the SARS-CoV-2 sub-lineages that are relevant to the epidemiological situation in the period under analysis through time based on data from GISAID. The study found that the XBB variant strain was dominant during the period from January to June 2023, accounting for approximately 57.63% of cases. It also provided valuable insights into the genomic characteristics of the Omicron subvariants and their potential impact on vaccine efficacy. |
| ***Strengths and Limitations*** | 19 | Discuss limitations of the study, taking into account sources of potential bias or imprecision. Discuss both direction and magnitude of any potential bias | First, the study is based on genomic data from GISAID, which may not be representative of the entire population, as it is based on samples that have been sequenced and shared by researchers and public health agencies. Therefore, the results of the study may not be generalizable to the entire population.  Second, the study covers the period from January to June 2023, which may not reflect the current epidemiological situation or the emergence of new variants. The study may not capture the full impact of the Omicron subvariants or other emerging variants that may arise in the future. |
| ***Interpretation*** | 20 | Give a cautious overall interpretation of results considering objectives, limitations, multiplicity of analyses, results from similar studies, and other relevant evidence | The study is based on genomic data from GISAID, which may not be representative of the entire population, and the study covers the period from January to June 2023, which may not reflect the current epidemiological situation or the emergence of new variants. Moreover, it also does not provide explicit information on the characteristics of study participants, missing data, or potential confounders, which may limit the ability to control for confounding or to generalize the results to other populations.  Therefore, a cautious interpretation of the results is warranted, and the results should be considered in the context of the study's limitations. It is also important to consider the results from similar studies and other relevant evidence when interpreting the results. Further research is needed to confirm the findings of this study and to assess the impact of the Omicron subvariants on vaccine efficacy and preparedness. |
| ***Generalizability*** | 21 | Discuss the generalisability (external validity) of the study results | The study is based on genomic data from GISAID, which is a global initiative to share genomic data of influenza viruses and the coronavirus responsible for COVID-19. The data available in GISAID may not be representative of the entire population, as it is based on samples that have been sequenced and shared by researchers and public health agencies. Therefore, the results of the study may not be generalizable to the entire population.  Additionally, the study covers the period from January to June 2023, which may not reflect the current epidemiological situation or the emergence of new variants. The study may not capture the full impact of the Omicron subvariants or other emerging variants that may arise in the future.  Furthermore, the study does not provide explicit information on the characteristics of study participants, missing data, or potential confounders, which may limit the ability to control for confounding or to generalize the results to other populations.  Therefore, the generalizability of the study results may be limited by the population and context in which the study was conducted, as well as the limitations of the study design. It is important to consider the limitations of the study when assessing the generalizability of the results and to interpret the results with caution. Further research is needed to confirm the findings of this study and to assess the generalizability of the results to other populations and contexts. |
| ***OTHER INFORMATION*** |  |  |  |
| **Funding** | 22 | Give the source of funding and the role of the funders for the present study and, if applicable, for the original study on which the present article is based | The study states that "No funding received for the work". Therefore, it appears that the present study did not receive any external funding. |

***N/A: Not available**

**Table S2. 10 questions for appraising qualitative research (CASP checklist)**

| ***Questions*** | ***HINT: Consider*** | ***Response*** |
| --- | --- | --- |
| 1. Was there a clear statement of the aims of the research? | - what was the goal of the research - why it was thought important its relevance | The study provides a clear overview of the purpose and scope of the analysis, which is to provide insights and strategic approaches for vaccine formulation and preparedness in response to the continuously emerging Omicron subvariants of SARS-CoV-2.  Therefore, it does provide a clear and relevant focus for the analysis. |
| 2. Is a qualitative methodology appropriate? | - If the research seeks to interpret or illuminate the actions and/or subjective experiences of research participants - Is qualitative research the right methodology for addressing the research goal | The methods section of the study describes the use of statistical analysis to estimate the transmission fitness advantage and the effective reproductive number (R_e_) of the SARS-CoV-2 sub-lineages that are relevant to the epidemiological situation in the period under analysis through time based on data from GISAID.  Based on this information, it appears that a quantitative methodology was used rather than a qualitative methodology. However, it is important to note that the specific methodology used may vary depending on the research question and objectives. |
| 3. Was the research design appropriate to address the aims of the research? | - If the researcher has justified the research design (e.g. have they discussed how they decided which method to use) | The study provides a clear overview of the purpose and scope of the analysis, which is to provide insights and strategic approaches for vaccine formulation and preparedness in response to the continuously emerging Omicron subvariants of SARS-CoV-2.  The methods section of the study describes the use of statistical analysis to estimate the transmission fitness advantage and the effective reproductive number (R_e_) of the SARS-CoV-2 sub-lineages that are relevant to the epidemiological situation in the period under analysis through time based on data from GISAID.  Based on this information, it appears that the research design was appropriate to address the aims of the research, as it involved the use of statistical analysis to investigate the emergence and spread of SARS-CoV-2 variants and the potential of next-gen vaccines to combat continuously emerging Omicron subvariants. |
| 4. Was the recruitment strategy appropriate to the aims of the research? | - If the researcher has explained how the participants were selected - If they explained why the participants they selected were the most appropriate to provide access to the type of knowledge sought by the study - If there are any discussions around recruitment (e.g. why some people chose not to take part) | The study does not provide any information on recruitment strategies, as the study did not involve human participants. Instead, the study used data from GISAID to estimate the transmission fitness advantage and the effective reproductive number (R_e_) of the SARS-CoV-2 sub-lineages that are relevant to the epidemiological situation in the period under analysis through time.  Therefore, recruitment strategies are not applicable to this study, as it did not involve human participants. |
| 5. Was the data collected in a way that addressed the research issue? | - If the setting for the data collection was justified - If it is clear how data were collected (e.g. focus group, semi-structured interview, etc.) - If the researcher has justified the methods chosen - If the researcher has made the methods explicit (e.g. for interview method, is there an indication of how interviews are conducted, or did they use a topic guide) - If methods were modified during the study. If so, has the researcher explained how and why - If the form of data is clear (e.g. tape recordings, video material, notes, etc.) - If the researcher has discussed saturation of data | The study describes the use of data from GISAID to estimate the transmission fitness advantage and the effective reproductive number (R_e_) of the SARS-CoV-2 sub-lineages that are relevant to the epidemiological situation in the period under analysis through time.  The study aimed to investigate the prevalence and impact of SARS-CoV-2 variants and to provide insights and strategic approaches for vaccine formulation and preparedness in response to the continuously emerging Omicron subvariants.  Based on this information, it appears that the data collected in the study were appropriate to address the research issue, as the study used data from GISAID to estimate the transmission fitness advantage and the effective reproductive number of the SARS-CoV-2 sub-lineages that are relevant to the epidemiological situation in the period under analysis through time. |
| 6. Has the relationship between research and participants been adequately considered? | - If the researcher critically examined their own role, potential bias and influence during (a) formulation of the research questions; (b) data collection, including sample recruitment and choice of location - How the researcher responded to events during the study and whether they considered the implications of any changes in the research design | The study does not provide any information on the involvement of human participants in the study. Instead, the study used data from GISAID to estimate the transmission fitness advantage and the effective reproductive number (R_e_) of the SARS-CoV-2 sub-lineages that are relevant to the epidemiological situation in the period under analysis through time.  Therefore, the relationship between research and participants is not applicable to this study, as it did not involve human participants. |
| 7. Have ethical issues been taken into consideration? | - If there are sufficient details of how the research was explained to participants for the reader to assess whether ethical standards were maintained - If the researcher has discussed issues raised by the study (e.g., issues around informed consent or confidentiality or how they have handled the effects of the study on the participants during and after the study) - If approval has been sought from the ethics committee | The study does not provide any information on ethical issues related to the study. However, the authors state that the study did not involve human participants and that the data used in the study are available from the corresponding author upon a reasonable request.  Therefore, it appears that ethical issues related to human participants are not applicable to this study, as it did not involve human participants. |
| 8. Was the data analysis sufficiently rigorous? | - If there is an in-depth description of the analysis process - If thematic analysis is used. If so, is it clear how the categories/themes were derived from the data - Whether the researcher explains how the data presented were selected from the original sample to demonstrate the analysis process - If sufficient data are presented to support the findings - To what extent contradictory data are taken into account - Whether the researcher critically examined their own role, potential bias and influence during analysis and selection of data for presentation | The study provides specific details on the statistical methods and sources used (scientific articles, software programs, and code).  Therefore, the data analysis was sufficiently rigorous. |
| 9. Is there a clear statement of findings? | - If the findings are explicit - If there is adequate discussion of the evidence both for and against the researcher’s arguments - If the researcher has discussed the credibility of their findings (e.g., triangulation, respondent validation, more than one analyst) - If the findings are discussed in relation to the original research question | The discussion and conclusion sections of the study summarize the findings of the work, highlight the importance of effective statistical analysis in tracking the emergence and spread of SARS-CoV-2 variants (e.g., XBB had an estimated 28% relative weekly growth advantage compared to other globally-circulating variants), and emphasize the need for global collaboration and rapid vaccine development and distribution to effectively combat emerging variants.  In addition, the study describes future recommendations, which could focus on (i) improving vaccine development and distribution infrastructure, increasing vaccine production capacity, and ensuring equitable access to vaccines for all populations; (ii) developing predictive models that can forecast the emergence and spread of new variants, which can inform public health policies and decision-making. |
| 10. How valuable is the research? | - If the researcher discusses the contribution the study makes to existing knowledge or understanding (e.g., do they consider the findings in relation to current practice or policy, or relevant research based literature - If they identify new areas where research is necessary - If the researchers have discussed whether or how the findings can be transferred to other populations or considered other ways, the research may be used | The study presents a comprehensive analysis of the prevalence and impact of SARS-CoV-2 variants, as well as the potential of next-gen vaccines to combat continuously emerging Omicron subvariants. The study also estimates the transmission fitness advantage and the effective reproductive number (R_e_) of the SARS-CoV-2 sub-lineages that are relevant to the epidemiological situation in the period under analysis through time.  Overall, the research provides valuable insights into the development of next-gen vaccines and the tracking of emerging variants, which can inform public health policies and strategies to combat the COVID-19 pandemic. |
